# Perioperative and prognostic implication of albumin‐bilirubin‐TNM score in Child‐Pugh class A hepatocellular carcinoma

**DOI:** 10.1002/ags3.12212

**Published:** 2018-09-27

**Authors:** Fuminori Sonohara, Suguru Yamada, Nobutake Tanaka, Masaya Suenaga, Hideki Takami, Masamichi Hayashi, Yukiko Niwa, Hiroyuki Sugimoto, Norifumi Hattori, Mitsuro Kanda, Chie Tanaka, Daisuke Kobayashi, Goro Nakayama, Masahiko Koike, Michitaka Fujiwara, Yasuhiro Kodera

**Affiliations:** ^1^ Department of Gastroenterological Surgery Nagoya University Graduate School of Medicine Nagoya Japan; ^2^ Department of Surgery Komaki City Hospital Komaki Japan

**Keywords:** ALBI‐TNM score, albumin‐bilirubin grade, hepatectomy, hepatocellular carcinoma

## Abstract

**Background and Aim:**

A reliable classification for predicting postoperative prognosis and perioperative risk of hepatocellular carcinoma (HCC) patients is required to make a precise decision for HCC treatment. In the present study, we assessed the perioperative and prognostic importance of indocyanine green (ICG) testing, tumor‐node‐metastasis (TNM) stage, albumin‐bilirubin (ALBI) grade, and ALBI‐TNM (ALBI‐T) score using consecutive resected HCC cases.

**Methods:**

Between 1998 and 2011, 273 consecutive patients who underwent primary and curative hepatectomy for HCC were identified. Among these 273 cases, 235 Child‐Pugh class A patients were enrolled in the present study.

**Results:**

Correlation analysis showed that the value of linear predictor for ALBI grade was significantly correlated with ICG 15‐minute retention rates (*r* = 0.51, *P* < 0.0001). Survival analysis for both recurrence‐free survival (RFS) and overall survival (OS) showed there were significant differences between the two groups stratified by stage or ALBI‐T score (stage, RFS:* P* = 0.01, OS:* P* = 0.003; ALBI‐T, RFS:* P* < 0.0001, OS:* P* < 0.0001). In addition, Cox proportional hazard model identified ALBI‐T score was a significant predictor for both RFS and OS (RFS,* P* = 0.001; OS,* P* = 0.004). Furthermore, ALBI‐T score could predict perioperative risk in hepatectomy such as longer operation time and excessive intraoperative blood loss.

**Conclusions:**

This study showed a robust association of ALBI‐T score with postoperative HCC patient survival and perioperative risk in hepatectomy. ALBI‐T score can be used as a simple and powerful tool for assessing HCC patients with further study.

## 1 INTRODUCTION

Hepatocellular carcinoma (HCC) is a lethal disease and the second leading cause of cancer death worldwide.^1^ For curative treatment of HCC, hepatectomy (hepatic resection) is a major and desirable strategy.^2^, ^3^, ^4^ However, even after curative hepatectomy, 80% of patients develop HCC recurrence in the remnant liver and 50% die within 5 years.^5^ Hetero chronological multiple HCC occurrences are generally associated with background hepatitis caused by virus, alcohol, and non‐alcoholic fatty liver disease^6^ whereas intrahepatic tumor metastasis is mainly attributed to invasiveness of primary loci and relatively early recurrence.^7^ According to these two types of hepatic recurrence, both background liver status and tumor factor of HCC should be considered to make a precise decision for HCC treatment.

Currently, the American Joint Committee on Cancer (AJCC)/Union for International Cancer Control (UICC) tumor‐node‐metastasis (TNM) staging system is commonly used for evaluating pretreatment tumor status, and Child‐Pugh (CP) classification is applied for evaluating background liver status.^8^ However, as previously mentioned, CP classification and the integrated scoring system using CP classification have several limitations as a result of including subjective values such as grading of ascites and hepatic encephalopathy.^9^ Indocyanine green (ICG) testing is usually carried out prior to HCC treatment for assessing background liver status especially in Asian countries and in several institutes in Europe.^9^, ^10^ Although ICG testing well reflects the liver function, this examination requires well‐organized preparation to obtain an accurate result such as pre‐examination bed‐rest whether or not using an ICG clearance meter that does not require multiple blood sampling. Hence, a simple and accurate way to evaluate both tumor and background liver status of HCC prior to treatment has been strongly desired.

Lately, albumin‐bilirubin (ALBI) grade^11^ and ALBI‐TNM (ALBI‐T) score^12^ have attracted clinicians’ attention as more convenient and precise methods to evaluate HCC and background liver. Although there are some important studies showing the prognostic impact of ALBI grade and ALBI‐T score on HCC treatment,^11^, ^12^ their actual significance to HCC surgery is still being considered. In the present study, we retrospectively assessed the usefulness of ICG testing, TNM stage of the Liver Cancer Study Group of Japan (LCSGJ),^13^ ALBI grade, and ALBI‐T score to predict HCC prognosis after hepatectomy and to evaluate the risk at hepatectomy by conducting a search of consecutive resected HCC cases from our institute.

## 2 METHODS

### 2.1 Patients enrolled in the present study

Between January 1998 and December 2012, 273 consecutive patients who underwent curative hepatectomy for HCC at the Department of Gastroenterological Surgery, Nagoya University Hospital (Nagoya, Japan) were identified. The 235 cases identified as CP class A were enrolled in this study. Written informed consent, as required by the Institutional Review Board, was obtained from all patients for use of the anonymized information.

### 2.2 Clinical examination and hepatectomy

Preoperative blood examination was carried out 1 or 2 days prior to surgery. Serum albumin (Alb) concentrations, total bilirubin (T‐bil), alpha fetoprotein (AFP), hepatitis C virus (HCV) antibody, hepatitis B virus (HBV) surface antigen concentrations and prothrombin time (PT) were preoperatively measured. ICG testing was carried out before surgery with 162 HCC cases (69%), and ICG 15‐min retention rates (ICG‐R15) were calculated. Indications for surgery and extent of hepatectomy were determined based on the size, number and location of HCC, presence of ascites, serum Alb and T‐bil concentrations, PT, computed tomography (CT) volumetry findings, and results of the ICG test.

During the hepatectomy, hepatic parenchyma was mainly dissected with a CUSA system (Valley Lab, Boulder, CO, USA) and a VIO soft‐coagulation system (ERBE Elektromedizin, Tübingen, Germany) used since 2007. Most patients underwent surgery using the intermittent Pringle maneuver, clamping the portal triad for 15 minutes each at 5‐minute intervals.^14^ In appropriate cases, the liver hanging maneuver^15^ and the Glissonean pedicle transection method^16^ were carried out both respectively and jointly. Resection was defined as curative when all gross tumors were completely removed; cases of incidentally found small lesions suspected to be HCC that were treated by radiofrequency therapy or microwave coagulation therapy during the surgery were regarded as curative cases.

Surgery‐related variables included operation time, intraoperative blood loss (IOBL), and requirement for intraoperative blood transfusion. Tumor‐related variables included tumor number and size, and postoperative pathological variables (tumor differentiation, serosal invasion, capsule formation, capsule infiltration, septal formation, vascular invasion, bile duct invasion, and surgical margin). Tumors were categorized as well/ moderately or poorly differentiated, whereas the other pathological variables were categorized as positive or negative, as described by the guidelines of the LCSGJ.^13^ We used the definition of the Clavien‐Dindo classification to assess postoperative ascites, pleural effusion, bile leakage, and surgical site infection and grade IIIa or greater was considered positive.^17^ As for postoperative liver failure, we referred to the grading by the International Study Group of Liver Surgery and, in the present study, grade B and C were considered positive.^18^

### 2.3 Follow up after surgery

After discharge, patients were followed up once per month for 3 months and every 3 months thereafter. Blood examination, including those for serum AFP and des‐gamma‐carboxy prothrombin, was carried out at every outpatient care visit, and dynamic contrast‐enhanced CT was done every 6 months. Patients with abnormal data or suspected lesions underwent further examinations, including contrast ultrasonography, magnetic resonance imaging with gadolinium‐ethoxybenzyl‐diethylenetriamine pentaacetic acid, CT with hepatic arterioportography, and/or positron emission tomography for the diagnosis of HCC recurrence.

### 2.4 Classifications

Albumin‐bilirubin score was calculated and patients were classified with the cut‐off points as previously reported.^11^ Linear predictor for ALBI grade was calculated with the following equation: linear predictor (xb) = (log_10_ T‐bil × 0.66) + (Alb × −0.085), where T‐bil is in μmol/L and Alb is in g/L. The cut‐off points for ALBI grade were as follows: grade 1, xb ≤ −2.60; grade 2, −2.60 < xb ≤ −1.39; grade 3, −1.39 < xb. TNM stage of each patient was defined with General Rules for the Clinical and Pathological Study of Primary Liver Cancer, Nationwide Follow‐Up Survey and Clinical Practice Guidelines by LCSGJ.^13^ T factor for TNM stage of LCSGJ is as shown in Table S1. ALBI‐T score was calculated using the following equation: ALBI‐T score = ALBI grade + TNM stage of LCSGJ − 2.^12^

### 2.5 Statistical analysis

All statistical analyses were carried out using R version 3.4.3 (https://www.r-project.org/). Continuous variables were expressed as medians (ranges) and compared using the Wilcoxon rank‐sum test, and categorical variables were compared using the chi‐squared test or Fisher's exact test, as appropriate. Recurrence‐free survival (RFS) was defined as the time between the curative resection of HCC and confirmation of recurrence. Overall survival (OS) was defined as the time between the operation and all‐cause death. Cox proportional hazards models were used to determine the risk factors associated with RFS and OS. Survival analysis based on the Kaplan‐Meier method and log‐rank tests was also carried out. The level of statistical significance was set at *P* < 0.05, which was obtained using two‐tailed tests.

## 3 RESULTS

### 3.1 Patient characteristics

In the present study, 235 CP class A HCC patients were enrolled, as other classes (B and C) of CP classification were rare in the patients who underwent hepatectomy and the class B cases were hypothesized to have worse prognosis than grade A.^19^ Patient demographic and clinical characteristics are shown in Table 1. Median patient follow‐up time for all cases was 44.1 months (range, 0.3‐194 months). At the end of the follow‐up period, 108 (46%) patients had died, and median duration from time of surgery to death in these cases was 32 months (range, 0.3 to 169 months). The number of patients who died within 90 days of their surgery was nine (3.8%), and there was no case of intraoperative death. Tumor recurrence in remnant liver occurred in 117 patients (81%), and 19 patients (13%) had distant metastasis without hepatic recurrence after surgery. Median time to postoperative recurrence or distant metastasis was 19 months (range, 0‐162 months). In nine patients (6%) information on the locations of tumor recurrence was missing.

**Table 1:** Characteristics of hepatocellular carcinoma patients in the present study (n = 235)

Characteristics		Value
Age (y)	Median (range)	65 (33–84)
Gender, n (%)	Male : Female	192 (82) : 43 (18)
Viral infection,^a^ n (%)	HBV : HCV : HBV + HCV : non‐HBV/HCV	66 (28) : 118 (50) : 4 (2) : 47 (20)
Liver damage classification,^b^ n (%)	A : B : C	175 (75) : 31 (13)
Albumin (g/dL)	Median (range)	4.0 (2.8–4.9)
Total bilirubin (mg/dL)	Median (range)	0.7 (0.2–2.4)
PT (%)	Median (range)	89.9 (40.1–138)
AFP (ng/mL)	Median (range)	17 (0.8–222 228)
Tumor size (cm)	Median (range)	3.5 (0.08–15)
Tumor multiplicity	Solitary : Multiple	178 (76) : 57 (24)
ICG‐R15 (%)	Median (range)	11.4 (1.6–35.2)
Stage,^c^ n (%)	I : II : III : IV	27 (12) : 122 (53): 54 (23): 28 (12)

AFP, alpha‐fetoprotein; HBV, hepatitis B virus; HCC, hepatocellular carcinoma; HCV, hepatitis C virus; ICG‐R15, retention rate of indocyanine green 15 min after dosage; n, number; PT, prothrombin time.

a Five cases with both HBV and HCV are included in each type.

b There are 29 cases without liver damage information.

c There are 4 cases without stage information.

### 3.2 Albumin‐bilirubin (ALBI) grade and ALBI‐TNM score in Child‐Pugh class A HCC patients

**Figure 1:**
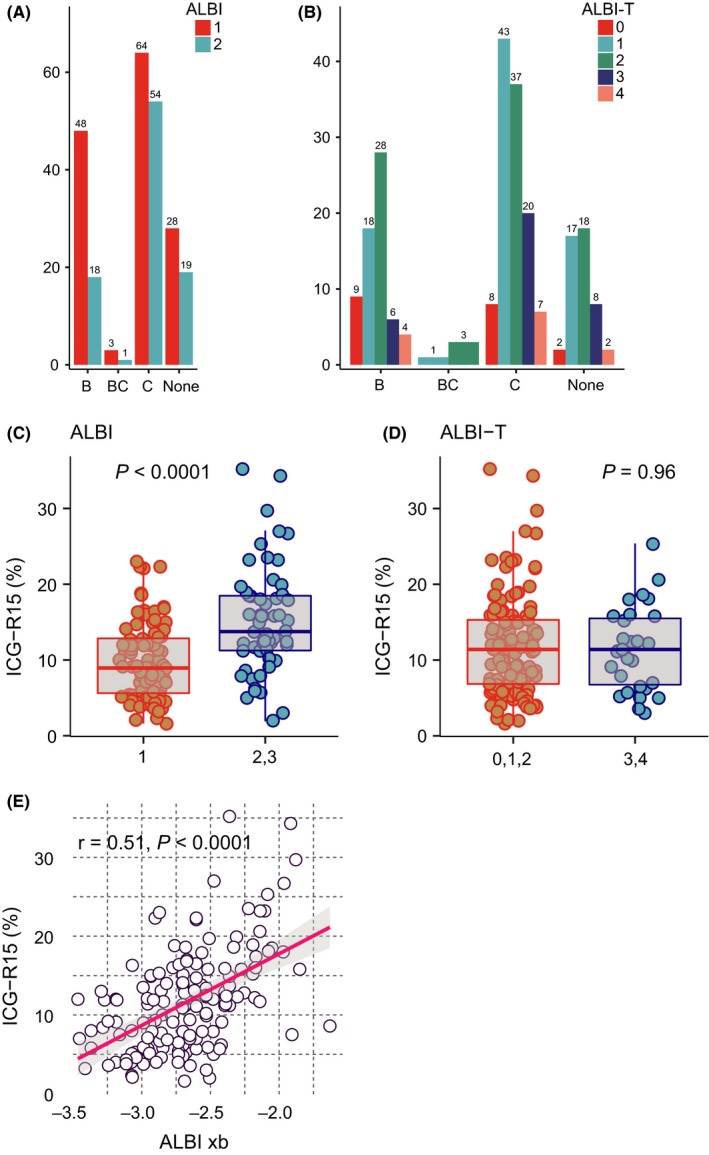
Albumin‐bilirubin (ALBI) grade and albumin‐bilirubin‐TNM (ALBI‐T) score in Child‐Pugh class A hepatocellular carcinoma (HCC) patients. A, Distribution of ALBI grade according to virus status. B, Distribution of ALBI‐T score according to virus status. C, Retention rate of indocyanine green 15 min after dosage (ICG‐R15) in ALBI grade 1 and ALBI grade 2. D, ICG‐R15 in ALBI‐T score 0, 1, 2 and 3, 4. E, Correlation between value of linear predictor for ALBI grade (ALBI xb) and ICG‐R15. Red solid line indicates linear regression line, and the greyish area indicates 95% confidence region of linear regression

Among 235 CP class A HCC patients, 142 (60%) patients were classified as ALBI grade 1 and 93 (40%) patients were grade 2 and there was no patient classified as grade 3. Histogram of ALBI grade based on hepatitis virus infection is shown in Figure 1A. In HCC patients with HBV, 47 patients (72%) were classified as grade 1 and the proportion of ALBI grade was significantly different between HBV and HCV patients (*P* = 0.007). Using ALBI‐T score, 231 informative cases with preoperative stage based on the guidelines of the LCSGJ were classified as follows: score 0; 19 (8%), score 1; 79 (34%), score 2; 86 (37%), score 3; 34 (15%), score 4; 13 (6%); and score 5, 0. Histogram of ALBI‐T score based on virus infection is shown in Figure 1B. The most dominant ALBI‐T score in patients with HBV was score 2 whereas score 1 was most frequently seen in patients with HCV (Figure 1B). HCC patients without any hepatitis virus had the same tendency of distribution in ALBI and ALBI‐T as patients with HCV (Figure 1A,B).

Distributions of clinicopathological features in the HCC patients according to ALBI grade and ALBI‐T score are shown in Tables S2 and S3. Proportion of patients with ICG‐R15 (<15 or ≥15) was significantly different between ALBI grade 1 and 2 (*P* < 0.0001) as well as Alb concentration (<3.5 or ≥3.5, *P* < 0.0001) and liver damage score (A or B, C, <0.0001, Table S2) defined by presence of ascites, T‐bil concentration, Alb concentration, ICG‐R15, and PT.^11^ As for ALBI‐T score, the proportion of patients was significantly different according to gender (*P* = 0.03), Alb concentration (*P* = 0.01), number of tumors (multiple or solitary, *P* < 0.0001), tumor size (<2 or ≥2 cm, *P* = 0.003), growth form (expansive or infiltrative, *P* = 0.001), serosal invasion (*P* = 0.003), vascular invasion (*P* < 0.0001), and stage (<III or ≥III, *P* < 0.0001, Table S3).

### 3.3 Association among ALBI grade, ALBI‐T score, and ICG‐R15

In 154 informative cases with ICG‐R15, a significant difference of ICG‐R15 value could be identified between ALBI grade 1 and ALBI grade 2 (*P* < 0.0001, Figure 1C) although there was no significant difference of ICG‐R15 between ALBI‐T 0,1,2 and ALBI‐T 3,4 (*P* = 0.96, Figure 1D). In addition, correlation analysis showed that the value of a linear predictor for ALBI grade (xb) was moderately correlated with ICG‐R15 (*r* = 0.51, *P* < 0.0001, Figure 1E). The significant correlation between ALBI grade and ICG‐R15 may be explained as both ALBI grade and ICG‐R15 well reflected the background hepatitis status, so both ALBI grade and ICG‐R15 had the same direction in CP class A patients.

### 3.4 Hepatocellular carcinoma prognosis stratified by ICG‐R15, stage, ALBI grade, and ALBI‐T score

**Figure 2:**
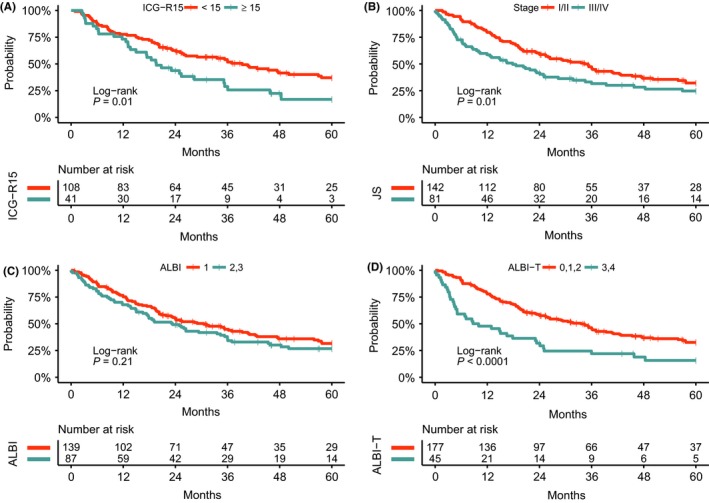
Survival analysis stratified by indocyanine green 15 min after dosage (ICG‐R15), stage, albumin‐bilirubin (ALBI) grade, and albumin‐bilirubin‐TNM (ALBI‐T) score. A, Recurrence‐free survival (RFS) analysis stratified by ICG‐R15. B, RFS analysis stratified by stage. C, RFS analysis stratified by ALBI. D, RFS analysis stratified by ALBI‐T

**Figure 3:**
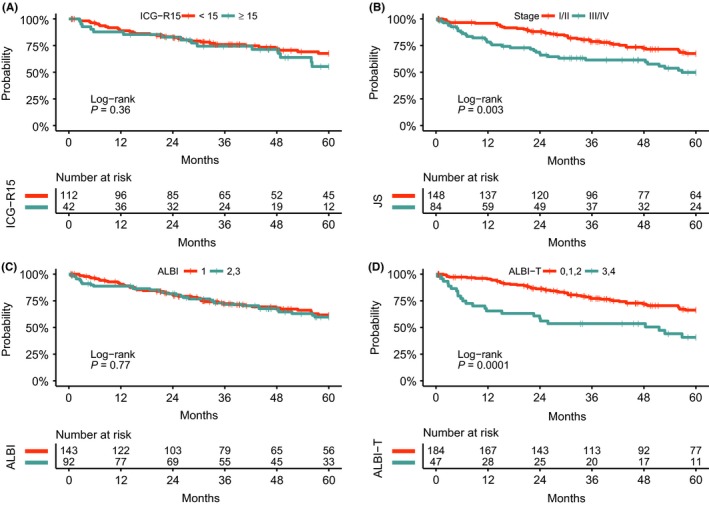
Survival analysis stratified by indocyanine green 15 min after dosage (ICG‐R15), stage, albumin‐bilirubin (ALBI) grade, and albumin‐bilirubin‐TNM (ALBI‐T). A, Overall survival (OS) analysis stratified by ICG‐R15. B, OS analysis stratified by stage. C, OS analysis stratified by ALBI. D, OS analysis stratified by ALBI‐T

First, to carry out a comparative prognostic analysis, the 235 CP class A cases with curative hepatectomy were divided into two groups according to ICG‐R15, stage, ALBI grade, and ALBI‐T score as follows: ICG‐R15 (<15 vs ≥15), stage (I/ II vs III/ IV), ALBI grade (1 vs 2), and ALBI‐T (0, 1, 2 vs 3, 4). Next, survival analysis stratified by ICG‐R15, stage, ALBI grade, and ALBI‐T score was carried out using Kaplan‐Meier analysis (Figures 2, 3). In both RFS and OS, there were significant differences between the two groups stratified by stage or ALBI‐T score (stage, RFS: *P* = 0.01, OS: *P* = 0.003; ALBI‐T, RFS: *P* < 0.0001, OS: *P* = 0.0001, Figures 2B,D and 3B,D). ICG‐R15 could also stratify the RFS of HCC cases with a statistical significance (*P* = 0.01, Figure 2A) but there was no significance in OS (*P* = 0.36, Figure 3A). Furthermore, RFS and OS analysis with the Cox proportional hazard models identified ALBI‐T score as a significant predictor for both RFS and OS (RFS, *P* = 0.001; OS, *P* = 0.004) as well as virus status (RFS, *P* = 0.02; OS, *P* = 0.01), ICG‐R15 (RFS, *P* = 0.003; OS, *P* = 0.01), liver damage (RFS, *P* = 0.002; OS, *P* = 0.005), tumor number (RFS, *P* = 0.004; OS, *P* = 0.005), differentiation (RFS, *P* = 0.02; OS, *P* = 0.004), serosal invasion (RFS, *P* < 0.0001; OS, *P* = 0.002), vascular invasion (RFS, *P* < 0.0001; OS, *P* = 0.0002), and stage (RFS, *P* = 0.02; OS, *P* = 0.03, Tables 2 and 3). As in our previous report, serosal and vascular invasions that were tumor factors diagnosed with resected specimens were a strong predictor for HCC prognosis.^20^ Among the features that can be presurgically obtained and the classifications using presurgical features, ALBI‐T score was able to separate CP class A cases into different prognoses both in RFS and OS better than other classifications or clinical features.

**Table 2:** Univariate analysis of recurrence‐free survival

Variables		HR	95% CI Low	95% CI High	*P*
Age (y)	≥65 vs <65	1.24	0.91	1.68	0.17
Gender	Male vs female	0.27	0.21	1.28	0.20
Virus infection	HCV vs others	0.70	0.51	0.95	0.02
Albumin (g/dL)	<3.5 vs ≥3.5	1.80	1.17	2.79	0.008
PT (%)	<70 vs ≥70	1.09	0.64	1.87	0.74
ICG‐R15 (%)	≥15 vs <15	0.53	1.25	2.88	0.003
Liver cirrhosis	(+) vs (−)	1.34	0.98	1.84	0.07
Liver damage	B or C vs A	1.98	1.28	3.06	0.002
Tumor number	Multiple vs solitary	0.60	0.43	0.85	0.004
Tumor size (cm)	≥2 vs <2	1.71	1.05	2.80	0.03
AFP (ng/mL)	≥20 vs <20	1.37	0.99	1.88	0.05
Differentiation	Poor vs well/moderate	0.52	0.30	0.90	0.02
Growth form	Infiltrative vs expansive	1.14	0.75	1.71	0.55
Formation of capsule	(−) vs (+)	1.31	0.94	1.84	0.11
Infiltration to capsule	(+) vs (−)	1.11	0.81	1.50	0.52
Septal formation	(−) vs (+)	0.99	0.71	1.36	0.93
Serosal invasion	(+) vs (−)	2.22	1.53	3.23	< 0.0001
Portal vein or hepatic vein invasion	(+) vs (−)	1.99	1.43	2.77	< 0.0001
Surgical margin	(+) vs (−)	1.00	0.62	1.61	1.00
Stage	III/IV vs I/II	1.47	1.07	2.01	0.02
ALBI grade	2,3 vs 1	1.22	0.90	1.67	0.20
ALBI‐T score	3,4,5 vs 0,1,2	1.85	1.28	2.67	0.001

AFP, alpha fetoprotein; ALBI, albumin‐bilirubin; ALBI‐T, albumin‐bilirubin‐TNM; CI, confidence interval; HCV, hepatitis C virus; HR, hazard ratio; ICG‐R15, indocyanine green 15‐min retention rate; PT, prothrombin time.

**Table 3:** Univariate analysis of overall survival

Variables		HR	95% CI Low	95% CI High	*P*
Age (y)	≥65 vs <65	1.67	1.14	2.46	0.009
Gender	Male vs female	1.23	0.73	2.06	0.44
Virus infection	HCV vs others	0.61	0.41	0.90	0.01
Albumin (g/dL)	<3.5 vs ≥3.5	1.68	0.98	2.87	0.06
PT (%)	<70 vs ≥70	1.26	0.66	2.39	0.48
ICG‐R15 (%)	≥15 vs <15	1.97	1.15	3.38	0.01
Liver cirrhosis	(+) vs (−)	1.38	0.94	2.05	0.10
Liver damage	B or C vs A	2.10	1.25	3.53	0.005
Tumor number	Multiple vs solitary	0.60	0.39	0.90	0.01
Tumor size (cm)	≥2 vs <2	1.73	0.90	3.33	0.10
AFP (ng/mL)	≥20 vs <20	1.70	1.14	2.53	0.009
Differentiation	Poor vs well/moderate	0.39	0.21	0.73	0.004
Growth form	Infiltrative vs expansive	1.29	0.79	2.10	0.31
Formation of capsule	(−) vs (+)	1.11	0.73	1.68	0.62
Infiltration to capsule	(+) vs (−)	0.98	0.67	1.44	0.93
Septal formation	(−) vs (+)	0.84	0.57	1.25	0.40
Serosal invasion	(+) vs (−)	2.04	1.30	3.20	0.002
Portal vein or hepatic vein invasion	(+) vs (−)	2.18	1.45	3.27	0.0002
Surgical margin	(+) vs (−)	1.49	0.87	2.55	0.15
Stage	III/IV vs I/II	1.55	1.05	2.28	0.03
ALBI grade	2,3 vs 1	1.40	0.95	2.06	0.09
ALBI‐T score	3,4,5 vs 0,1,2	1.94	1.24	3.02	0.004

AFP, alpha fetoprotein; ALBI, albumin‐bilirubin; ALBI‐T, albumin‐bilirubin‐TNM; CI, confidence interval; HCV, hepatitis C virus; HR, hazard ratio; ICG‐R15, indocyanine green 15‐min retention rate; PT, prothrombin time.

### 3.5 Association of ALBI and ALBI‐T with perioperative risk in hepatectomy

**Figure 4:**
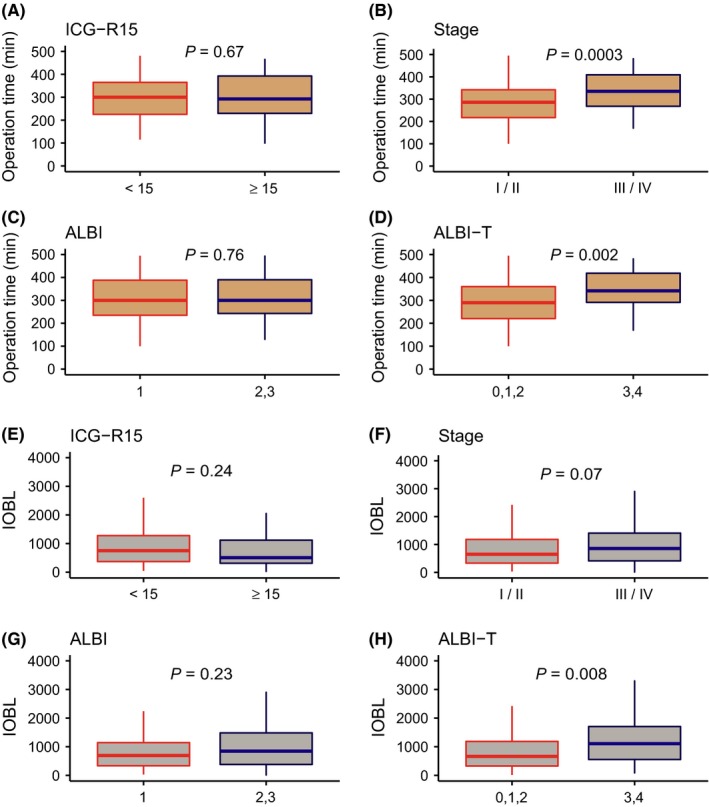
Operation time and intraoperative blood loss (IOBL) according to indocyanine green 15 min after dosage (ICG‐R15), stage, albumin‐bilirubin (ALBI) grade, and albumin‐bilirubin‐TNM (ALBI‐T) score. A, Operation time according to ICG‐R15. B, Operation time according to stage. C, Operation time according to ALBI grade. D, Operation time according to ALBI‐T. E, IOBL according to ICG‐R15. F, IOBL according to stage. G, IOBL according to ALBI. H, IOBL according to ALBI‐T

We also investigated the impact of ICG‐R15, stage, ALBI grade, and ALBI‐T score in CP class A patients to operation time, IOBL, and rate of transfusion during hepatectomy (Figure 4). ALBI‐T score 3, 4 showed significantly longer operative time (*P* = 0.002, Figure 4D) and significantly more IOBL than score 0, 1, 2 (*P* = 0.008, Figure 4H). Incidence of carrying out blood transfusion during hepatectomy was significantly higher in ALBI grade 2 cases (32/54 cases) than in grade 1 (24/110, *P* = 0.002), whereas there was no significant difference according to ALBI‐T (*P* = 0.13), ICG‐R15 (*P* = 0.83), and stage (*P* = 0.17). In addition, we assessed major complications after hepatectomy stratified by ICG‐R15, stage, ALBI grade, and ALBI‐T score (Table S4). According to this analysis, stage was associated with pleural effusion (*P* < 0.0001); ALBI grade was associated with persistent ascites (*P* = 0.002); and ALBI‐T score was associated with both ascites and pleural effusion (*P* = 0.04 and 0.0003). Consequently, these results indicate that ALBI‐T score has a capability of predicting operation time, IOBL, and postoperative complications preoperatively as well as predicting postoperative prognosis.

## 4 DISCUSSION

In this study, we evaluated perioperative clinical importance of ICG‐R15, stage, ALBI grade, and ALBI‐T score in CP class A patients who underwent hepatectomy for HCC. Because most of the patients who undergo hepatectomy are CP class A, we focused only on CP class A patients in the present study. Our findings suggested that ALBI grade correlates well with ICG‐R15 reflecting the background liver function. In addition, ALBI‐T score was a robust predictor for both RFS and OS in CP class A patients after hepatectomy. Furthermore, ALBI‐T score or ALBI grade was significantly associated with operation time, IOBL, and need for intraoperative transfusion. Consequently, ALBI‐T score is capable of assessing the preoperative risk and patients’ prognosis after hepatectomy. Currently, thanks to achievements made by our predecessors, the safety and feasibility of hepatectomy have been greatly improved. However, a report by the National Clinical Database group in Japan showed that the rate of mortality at hepatectomy and at 30 days postoperatively was still 2.3% and 1.3%, respectively, in 2016.^21^ We believe that even among patients diagnosed with relatively well‐preserved hepatic function (CP class A), some might be better off undergoing non‐surgical treatment. We believe that precise evaluation prior to surgery is essential to achieve patients’ maximum benefit. Thus, ALBI grade and ALBI‐T are potentially capable of contributing to precision treatment for HCC patients.

A major obstacle for HCC treatment is the high frequency of recurrence even after complete resection or liver transplantation.^22^ The poor prognosis and high frequency of HCC recurrence is associated with both tumor factors and background liver status.^23^ To deliver a desired precision treatment to HCC patients, estimating the prognosis of patients planning to undergo hepatectomy is essential. Furthermore, hepatectomy has a potential risk of massive bleeding requiring blood transfusion that causes severe complications such as hepatic failure after surgery.^24^ To avoid perioperative fatality, evaluating background liver status and developing an appropriate strategy are also crucial. Thus, in terms of the unique aspects of prognosis and operative risk of HCC, we compared ICG‐R15, stage, ALBI grade, and ALBI‐T score and finally shed light on the superiority of ALBI‐T comprising both tumor features and background liver status.

Tumor staging is generally needed to determine the patients’ survival probability after treatment, decide the most appropriate therapy, and enable an objective comparison among the outcomes of cancer research. Furthermore, it should allow us to predict the prognosis of resected cancer cases and individual treatment risk. For these reasons, staging systems should separate patients into groups with homogeneous prognosis, and serve to select the appropriate treatment.^25^ The AJCC/UICC TNM staging system for HCC incorporates tumor size and local invasiveness such as vascular invasion, and number of tumor nodules as well as lymph node and distant metastasis.^8^ As for various types of neoplasms, the TNM staging system is a reliable outcome predictor, but prognostic modeling in HCC is more complex than those in other gastrointestinal cancers. There are several classifications of HCC such as the Groupe d'Etude et de Traitement du Carcinome Hépatocellulaire prognostic classification,^26^ Cancer of the Liver Italian Program,^27^ Barcelona Clinic Liver Cancer staging,^28^ the Chinese University Prognostic Index,^29^ and staging according to the guidelines of the LCSGJ.^13^ However, none of these classifications has received universal acceptance.^30^ One of the reasons why HCC staging is difficult is its characteristic recurrence pattern: intrahepatic metastasis and multicentric occurrence.^23^ Therefore, prognostic classification for HCC should be related to both tumor factor and background liver status.

ALBI grade was originally developed by Johnson et al^11^ as a simple predictive model for HCC prognosis incorporating serum T‐bil and serum Alb concentrations only. This model can eliminate non‐objective factors such as presence of hepatic encephalopathy and ascites used by CP classification. The authors included HCC patients with several treatment modalities and demonstrated that ALBI grade was able to stratify the OS of 525 CP A and B patients who underwent hepatectomy. In addition, Hiraoka et al^12^ recently reported that ALBI‐T score has a better ability of predicting HCC prognosis than other classifications considering both tumor and background liver status. Consistent with previous reports, in CP class A patients who underwent hepatectomy for HCC, for the first time we showed that ALBI‐T score could stratify both RFS and OS more effectively than single ALBI grade, stage, and ICG‐R15. Interestingly, ALBI‐T score was superior to the LCSGJ staging system in stratification of HCC prognosis. Prognostic analysis in the present study showed that hepatic function assessed by both ICG‐R15 and ALBI grade was able to stratify RFS to some extent, but could not stratify OS very well. According to these results, we hypothesized that OS of CP class A patients with resectable HCC is mainly affected by tumor status before the patients suffer from late‐stage liver failure or multiple heterochronic recurrences. Thus, ALBI‐T score, which is a combination of ALBI grade and LCSGJ staging, could stratify HCC RFS more effectively than single classifications.

Hepatectomy has more potential risks for excessive IOBL than other types of gastrointestinal surgery as a result of anatomy and histology.^31^ Post‐surgical hepatic failure should always be considered prior to hepatectomy.^32^ Tumor factors such as size, vascular invasion, and bile duct invasion may prevent the surgeon from simple resection. Furthermore, in normal soft hepatic tissue, it is much easier to distinguish small vessels in hepatic parenchyma that should be precisely treated than in relatively fibrous hard tissue even if it has not yet reached liver cirrhosis.^33^ Thus, background liver status as well as HCC tumor itself should be accurately estimated and classified to minimize the potential risk in hepatectomy and to identify the individual benefit of surgery. The current study showed that ALBI‐T score was significantly associated with both operation time and IOBL and that ALBI grade was associated with intraoperative transfusion. With a further validation study, these characteristics of ALBI grade and ALBI‐T score could be useful for surgeons preparing for a hepatectomy.

The present study was able to show a significant association between ALBI‐T score and operation time, IOBL, and postoperative prognosis in CP class A patients. However, there are several inherent limitations in this study. First, this study was based on retrospective single‐institutional clinical information. The HCC patients enrolled in this study were from Japan only, and it is well known that HCC from different regions has a different etiology and prognosis. In addition, the screening system for HCC in Japan is well established and relatively small HCC can be frequently treated by hepatectomy. Thus, the ALBI‐T might only be suitable for evaluating patients from the same country as it comprises the stage defined by guidelines of LCSGJ. Confirmation of the capability of this model as a perioperative risk predictor with HCC patients from other regions is crucial in order to apply this model worldwide in the future. Second, as a result of the limitations of the information availability, we were not able to carry out the analysis specific to each hepatitis etiology. It might be better to assess ALBI and ALBI‐T performance in the same background hepatitis, as non‐viral hepatitis and associated HCC are becoming more common especially in developed countries.^34^, ^35^

In conclusion, the present study showed robust association of ALBI‐T score with perioperative risks of hepatectomy and postoperative patient survival in CP class A patients who underwent hepatectomy for HCC. ALBI‐T score is a simple and powerful tool for estimating both patient's tumor factor and background liver status simultaneously. With further study, we could use ALBI‐T score as a convenient way to assess HCC patients and deliver a more precise treatment to individual HCC patients.

## DISCLOSURE

Conflicts of Interest: Authors declare no conflicts of interests for this article.

Author Contributions: Conception and design: FS, SY, HS; Administrative support: YK, SY; Provision of study materials and patients: FS, NT, MS, HT, MH, YN, SY, HS, NH, MK, CT, DK, GN, MK, MF; Collection and assembly of data: FS, NT, SY, HS; Manuscript writing: FS; Final approval of manuscript: all authors.
