# Supplementary material for: Perioperative and prognostic implication of albumin‐bilirubin‐TNM score in Child‐Pugh class A hepatocellular carcinoma
Source: Ann Gastroenterol Surg. 2018 Sep 27;3(1):65–74. doi: 10.1002/ags3.12212 (PMC6345730; doi:10.1002/ags3.12212)
Supplement: Supplementary file 1 [file AGS3-3-65-s001.docx]

**Supplementary materials**

**Perioperative and prognostic implication of Albumin-Bilirubin-TNM Score in Child-Pugh Class A Hepatocellular Carcinoma**

Authors

Fuminori Sonohara, MD, PhD^1^; Suguru Yamada, MD, PhD^1^*; Hiroyuki Sugimoto, MD, PhD^2^*; Masaya Suenaga, MD, PhD^1^; Hideki Takami, MD, PhD^1^; Masamichi Hayashi, MD, PhD^1^; Tsutomu Fujii, MD, PhD^3^; Yasuhiro Kodera, MD, PhD^1^

* Corresponding authors

Affiliations

1. Department of Gastroenterological Surgery, Nagoya University Graduate School of Medicine, Nagoya, Japan
2. Department of Surgery, Komaki City Hospital, Komaki, Japan
3. Department of Surgery and Science, Graduate School of Medicine and Pharmaceutical Sciences, University of Toyama

Contents:

Supplementary Table S1

Definition of T factor for TNM stage of Liver Cancer Study Group of Japan

Supplementary Table S2

Clinical features stratified by ALBI grade

Supplementary Table S3

Clinical features stratified by ALBI-T grade

Supplementary Table S4

Postoperative complications according to ICG-R15, stage, ALBI grade, and ALBI-T score (n = 235).

| **Supplementary Table S1.** Definition of T factor for TNM stage of Liver Cancer Study Group of Japan | | | | |
| --- | --- | --- | --- | --- |
|  | T1 | T2 | T3 | T4 |
| Factor |  |  |  |  |
| Tumor number, solitary | All matched | Two matched | One matched | Neither matched* |
| Tumor size, <2 cm |  |  |  |  |
| No invasion to portal vein, hepatic vein, nor bile duct |  |  |  |  |
| * Tumor rupture should be treated as T4. |  |  |  |  |

| **Supplementary Table S2.** Clinical features stratified by ALBI grade | | |  |  |
| --- | --- | --- | --- | --- |
|  |  | ALBI grade | |  |
| Variables | | 1 | 2,3 | *P* |
| Age (years) | < 65 | 75 | 41 | 0.30 |
|  | ≥ 65 | 68 | 51 |  |
| Sex | Female | 23 | 20 | 0.36 |
|  | Male | 120 | 72 |  |
| Virus infection | HCV | 67 | 55 | 0.07 |
|  | Others | 76 | 37 |  |
| Albumin (g/dL) | ≥ 3.5 | 143 | 61 | < 0.0001 |
|  | < 3.5 | 0 | 31 |  |
| PT (%) | ≥ 70 | 127 | 87 | 0.20 |
|  | < 70 | 16 | 5 |  |
| ICG-R15 (%) | < 15 | 81 | 31 | < 0.0001 |
|  | ≥ 15 | 15 | 27 |  |
| Liver cirrhosis | Negative | 91 | 51 | 0.39 |
|  | Positive | 47 | 35 |  |
| Liver damage | A | 118 | 57 | < 0.0001 |
|  | B or C | 8 | 23 |  |
| Tumor number | Solitary | 111 | 67 | 0.50 |
|  | Multiple | 32 | 25 |  |
| Tumor size (cm) | < 2 | 25 | 11 | 0.30 |
|  | ≥ 2 | 112 | 79 |  |
| AFP (ng/mL) | < 20 | 70 | 45 | 0.84 |
|  | ≥ 20 | 61 | 43 |  |
| Differentiation | Well or moderate | 130 | 82 | 1.00 |
|  | Poorly | 10 | 6 |  |
| Growth form | Expansive | 120 | 72 | 0.21 |
|  | Infiltrative | 19 | 19 |  |
| Formation of capsule | Positive | 97 | 58 | 0.56 |
|  | Negative | 45 | 33 |  |
| Infiltration to capsule | Negative | 66 | 50 | 0.23 |
|  | Positive | 76 | 40 |  |
| Septal formation | Positive | 88 | 58 | 0.94 |
|  | Negative | 50 | 31 |  |
| Serosal invasion | Negative | 103 | 63 | 0.73 |
|  | Positive | 26 | 19 |  |
| Portal vein or hepatic vein invasion | Negative | 103 | 64 | 0.80 |
|  | Positive | 37 | 26 |  |
| Surgical margin | Negative | 111 | 74 | 0.29 |
|  | Positive | 19 | 7 |  |
| Stage | < III | 90 | 58 | 1.00 |
|  | ≥ III | 51 | 33 |  |
| Abbreviations: HCV, hepatitis C virus; PT, prothrombin time; ICG-R15, Indocyanine green 15-min retention rate; AFP, alpha fetoprotein; ALBI, albumin-bilirubin. | | | | |

| **Supplementary Table S3.** Clinical features stratified by ALBI-T grade | | |  |  |
| --- | --- | --- | --- | --- |
|  |  | ALBI-T score | |  |
| Variables | | 0,1,2 | 3,4 | *P* |
| Age (years) | < 65 | 91 | 23 | 1.00 |
|  | ≥ 65 | 93 | 24 |  |
| Sex | Female | 40 | 3 | 0.03 |
|  | Male | 144 | 44 |  |
| Virus infection | HCV | 92 | 27 | 0.45 |
|  | Others | 92 | 20 |  |
| Albumin (g/dL) | ≥ 3.5 | 165 | 35 | 0.01 |
|  | < 3.5 | 19 | 12 |  |
| PT (%) | ≥ 70 | 168 | 42 | 0.90 |
|  | < 70 | 16 | 5 |  |
| ICG-R15 (%) | < 15 | 88 | 22 | 1.00 |
|  | ≥ 15 | 33 | 9 |  |
| Liver cirrhosis | Negative | 113 | 28 | 0.84 |
|  | Positive | 64 | 18 |  |
| Liver damage | A | 140 | 33 | 0.07 |
|  | B or C | 20 | 11 |  |
| Tumor number | Solitary | 159 | 15 | < 0.0001 |
|  | Multiple | 25 | 32 |  |
| Tumor size (cm) | < 2 | 35 | 0 | 0.003 |
|  | ≥ 2 | 145 | 45 |  |
| AFP (ng/mL) | < 20 | 94 | 19 | 0.14 |
|  | ≥ 20 | 77 | 26 |  |
| Differentiation | Well or moderate | 171 | 39 | 0.13 |
|  | Poorly | 10 | 6 |  |
| Growth form | Expansive | 159 | 31 | 0.001 |
|  | Infiltrative | 22 | 16 |  |
| Formation of capsule | Positive | 122 | 31 | 1.00 |
|  | Negative | 61 | 16 |  |
| Infiltration to capsule | Negative | 92 | 22 | 0.77 |
|  | Positive | 90 | 25 |  |
| Septal formation | Positive | 114 | 31 | 0.63 |
|  | Negative | 65 | 14 |  |
| Serosal invasion | Negative | 138 | 27 | 0.003 |
|  | Positive | 28 | 17 |  |
| Portal vein or hepatic vein invasion | Negative | 160 | 6 | < 0.0001 |
|  | Positive | 22 | 40 |  |
| Surgical margin | Negative | 146 | 38 | 1.00 |
|  | Positive | 21 | 5 |  |
| Stage | < III | 148 | 0 | < 0.0001 |
|  | ≥ III | 36 | 47 |  |
| Abbreviations: HCV, hepatitis C virus; PT, prothrombin time; ICG-R15, Indocyanine green 15-min retention rate; AFP, alpha fetoprotein; ALBI-T, albumin-bilirubin-TNM. | | | | |

| **Supplementary Table S4.** Postoperative complications according to ICG-R15, stage, ALBI grade, and ALBI-T score (n = 235).* | | | | | | | | | | | | | | | | |
| --- | --- | --- | --- | --- | --- | --- | --- | --- | --- | --- | --- | --- | --- | --- | --- | --- |
|  |  | ICG-R15^†^ | | |  | Stage^‡^ | | |  | ALBI | | |  | ALBI-T | | |
|  |  | < 15 | ≥ 15 | *P* |  | I/II | III/IV | *P* |  | 1 | 2,3 | *P* |  | 0,1,2 | 3,4 | *P* |
| Ascites | - | 92 | 30 | 0.16 |  | 123 | 62 | 0.22 |  | 122 | 65 | 0.002 |  | 152 | 33 | 0.04 |
|  | + | 17 | 11 |  |  | 24 | 19 |  |  | 17 | 27 |  |  | 29 | 14 |  |
| Pleural effusion | - | 96 | 34 | 0.43 |  | 137 | 59 | < 0.0001 |  | 124 | 75 | 0.12 |  | 164 | 32 | 0.0003 |
|  | + | 13 | 7 |  |  | 10 | 22 |  |  | 15 | 17 |  |  | 17 | 15 |  |
| Bile leakage | - | 98 | 37 | 1.00 |  | 132 | 73 | 1.00 |  | 124 | 84 | 0.66 |  | 163 | 42 | 1.00 |
|  | + | 11 | 4 |  |  | 15 | 8 |  |  | 15 | 8 |  |  | 18 | 5 |  |
| SSI | - | 90 | 35 | 0.81 |  | 121 | 65 | 0.72 |  | 113 | 76 | 0.86 |  | 151 | 35 | 0.20 |
|  | + | 19 | 6 |  |  | 26 | 16 |  |  | 26 | 16 |  |  | 30 | 12 |  |
| Liver failure | - | 105 | 39 | 0.67 |  | 140 | 73 | 0.17 |  | 132 | 84 | 0.29 |  | 171 | 42 | 0.20 |
|  | + | 4 | 2 |  |  | 7 | 8 |  |  | 7 | 8 |  |  | 10 | 5 |  |
| * There are 4 cases without information of complications. ^†^ ICG testing was performed with 150 cases. ^‡^ There are 3 cases without stage information.  Abbreviations: ICG-R15, Indocyanine green 15-min retention rate; ALBI, albumin-bilirubin; ALBI-T, albumin-bilirubin-TNM; SSI, surgical site infection. | | | | | | | | | | | | | | | | |
